# Anxiety, Insomnia, and Memory Impairment in Metabolic Syndrome Rats Are Alleviated by the Novel Functional Ingredients from *Anacardium occidentale*

**DOI:** 10.3390/antiox11112203

**Published:** 2022-11-07

**Authors:** Pratthana Srichomphu, Jintanaporn Wattanathorn, Wipawee Thukham-mee, Supaporn Muchimapura

**Affiliations:** 1 Department of Physiology and Graduate School (Neuroscience Program), Faculty of Medicine, Khon Kaen University, Khon Kaen 40002, Thailand; 2 Research Institute for High Human Performance and Health Promotion, Khon Kaen University, Khon Kaen 40002, Thailand

**Keywords:** anxiety, insomnia, memory, *Anacardium occidentale*, metabolic syndrome

## Abstract

Despite an increase in the coexistence of metabolic syndrome (MetS) and psychological disorders, together with their great impact on socio-economic burdens, no protective strategies that focus on these situations are available. Due to the role of oxidative stress in the pathophysiology of metabolic syndrome (MetS) and psychological disorders, we hypothesized that substances possessing antioxidant activity such as the novel functional ingredients from *Anacardium occidentale* (AO) could mitigate common psychological disorders in MetS rats. Male Wistar rats, weighing 200–250 g, were induced with MetS through a 12-week high-fat and high-cholesterol diet (HFHC). Then, they were given AO orally via a gastric gavage needle at doses of 1, 10 and 100 mg/kg BW for 14 days. Spatial memory, anxiety, depression, and sleep behaviors, together with changes in oxidative stress status and neurotransmitters, were assessed. All doses of AO significantly improved memory, anxiety, and sleep, together with the suppression of oxidative stress, AChE, and GABA-T in the cerebral cortex and hippocampus. These results suggest the protective effect of AO against anxiety, insomnia, and memory impairment that coexist with the MetS condition via an improvement in oxidative stress and the functions of the cholinergic and GABAergic systems. However, this benefit requires clinical confirmation.

## 1. Introduction

Currently, a rapid increase in the global prevalence of non-communicable diseases such as metabolic syndrome (MetS) and psychiatric disorders is taking place, especially of depression, anxiety, sleep problems, and dementia. They are regarded as major public health challenges [1,2,3,4,5]. The comorbidity and bidirectional associations between MetS and psychiatric disorders have been reported [6,7,8,9,10,11]. It has been reported that the coexistence of MetS and psychiatric disorders such as anxiety, depression, insomnia, and memory impairment take a huge share of the healthcare budget, so these conditions have to be be considered [12,13,14,15]. Despite this great impact on socio-economic burdens, most psychological disorder treatments in MetS have been neglected. In addition, no preventive strategies against the common psychiatric disorders are available. Therefore, a novel strategy for protecting and mitigating against the psychiatric disorders mentioned earlier is required.

Accumulative lines of evidence have revealed that an elevation in oxidative stress can attack many organelles, giving rise to many deleterious effects. In the brain, it can induce lipid peroxidation, giving rise to a reduction in membrane fluidity, protein destruction, and the inactivation of receptors, enzymes, and ion channels. Furthermore, this change can also alter neurotransmitters’ balance, neuronal function, and brain activity [16,17,18,19,20]. The aforementioned changes, in turn, induce psychiatric disorders. Moreover, it has been demonstrated that oxidative stress plays a crucial role not only in the pathogenesis of psychiatric disorders but also in the pathogenesis of metabolic syndrome [21,22]. The coexistence of brain disorders and MetS can exacerbate the excess oxidative stress and lipid peroxidation, leading to greater brain damage and dysfunction, and the substances possessing antioxidant effects can mitigate these deleterious effects [23,24,25]. This information raises the possibility that substances possessing antioxidant effects can also mitigate the psychiatric disorders in the MetS condition.

Accumulative lines of evidence have demonstrated that polyphenol-enriched substances possessing strong antioxidant activity [26,27] have the potential to become medicaments in the field of mental health and metabolic syndrome [28,29]. Therefore, the beneficial effect of polyphenol-enriched functional ingredients has gained attraction. A recent study reported the antioxidant activity of dietary fiber, and this activity is based on the polyphenol that is bound to a polysaccharide complex [30]. In addition, dietary fiber consumption also decreases psychiatric disorders such as anxiety, depression, insomnia, and memory impairment [31,32,33,34,35,36]. Owing to these beneficial effects of polyphenol and dietary fiber, and the synergistic effect concept, the protective effects against psychiatric disorder mentioned earlier of the polyphenol- and dietary fiber-enriched functional ingredients have gained much attention.

*Anacardium occidentale* L. or cashew, an exotic and new economic crop in Thailand, has been used for a long time in traditional folklore. Its leaf is rich in phenolic compounds, tannin, vitamin C, carotenoids, and organic acids [37]. *A. occidentale* leaf possesses higher antioxidant activity than coconut, sweet orange, lemon, and papaya leaf [38]. It also exhibits an antiinflammatory effect [39]. In Thailand, an apple pulp of *A. occidentale* has been widely used in the juice production industry. Due to the continually increasing efforts to reduce food waste, a cashew apple pomace (derived from the squeezed cashew apple pulp) has been prepared as dietary fiber [40]. Owing to the beneficial effects of the polyphenol substance and dietary fiber on psychiatric disorders, together with the synergistic effect interaction concept mentioned earlier, we hypothesized that the polyphenol- and dietary fiber-enriched functional ingredient should mitigate psychiatric disorders in the MetS condition. Thus, anxiolytic, antidepression, sleep induction, and memory-enhancing effects were determined in MetS rats. Furthermore, the possible underlying mechanisms were also investigated.

## 2. Materials and Methods

### 2.1. Preparation of the Functional Ingredient (AO)

Both leaves and pomace of cashew or *Anacardium occidentale* Linn (var. Kaopayam) were obtained from Srisuphaluck Company, Phuket province, Thailand, during September 2018. After an authentication by the Taxonomist of the Department of Agriculture, Ministry of Agriculture and Cooperatives, the herbarium was deposited at Integrative Complementary Alternative Medicine Research Center in Research Institute for High Human Performance and Health Prevention, Khon Kaen University. A water extract of cashew or *A. occidentale* leaves was mixed with a cashew apple pomace-derived dietary fiber at an appropriate ratio that provides a combination index of antioxidant activity less than zero [41]. The detail of preparation was under the petty patent registration process, and the detail is mentioned elsewhere [42]. The developed functional ingredient contained polyphenolic compounds and flavonoids at the concentrations of 4.51 ± 0.14 µg gallic acid equivalent (GAE)/mg extract and 2.98 ± 0.27 µg quercetin/mg extract, respectively. The fingerprint chromatogram analysis revealed that the main flavonoids presented in AO were gallic acid, quercetin, and rutin at the concentrations of 42.91 ± 0.37 µg GAE/50 mg extract, 0.68 ± 0.40 µg Quercetin equivalents(QE)/50 mg extract, and 0.34 ± 0.04 µg rutin/50 mg extract, respectively. The fingerprint chromatogram of this functional ingredient is shown in Supplementary Materials (Figure S1).

### 2.2. Induction of Metabolic Syndrome (MetS)

It was previously reported that chronic high-fat and high-cholesterol diet (HFHC) could induce MetS. Therefore, we induced MetS by using HFHC according to formula of Panchal and coworkers [43]. It was revealed that normal diet or chow diet consisted of fat 4.5%, protein 24%, whereas HFHC consisted of fructose 17.5%, fat 20%, carbohydrate 35%, and protein 22%. In addition, a lard oil (pig oil) was also served as a fat source in this formula in order to induce foam cell formation and atherosclerosis [44]. In brief, a normal diet of 800 g was ground and mixed with 100 g of egg yolk and 100 g of pig oil. The obtained mixture was homogenized and rat diet was prepared and dried at 60 °C. The animals were fed with the prepared HFHC for 12 weeks. Only the rats with the MetS characteristics, including obesity, insulin resistance, and hypertension, were selected for further study.

### 2.3. Experimental Animals and Protocols

Male Wistar rats weighing 200–250 g were purchased from Nomura Siam International, Thailand. They were housed in groups of 4 per cage in standard metal cages at 22 ± 2 °C on 12:12 h light–dark cycle. All animals were given access to food and water ad libitum. The experiments were performed to minimize animal suffering and the experimental protocols were approved by the Institutional animal care and the animal ethics committee of Khon Kaen University, based on the ethics of animal experimentation of national research council of Thailand (ACUC-KKU-69/2559). All animals were randomly divided into 8 groups of 6 each as shown below, after one week of acclimatization.

Group I Naïve intact or control group; rats in this group received no treatment.
Group II Vehicle treated group; all rats in this group were fed with high-fat, high-cholesterol diet (HFHC) to induce metabolic syndrome (MetS) and received vehicle treatment.
Group III High-fat, high-cholesterol diet (HFHC) plus vitamin C-treated group. In this group, MetS rats were induced by HFHC and received vitamin C, a well-known antioxidant. This group also served as positive control owing to its antioxidant activity and its positive effect on stress-related disorders such as anxiety, depression [45], and memory [46].
Group IV High-fat, high-cholesterol diet (HFHC) plus standard drugs for treating anxiety, depression, or memory impairment. This group also served as positive control treated group. During the assessments of anxiolytic and sleep induction effects, benzodiazepine was served as a standard drug, whereas fluoxetine and donepezil were used as standard drugs for antidepression, and memory performance assessments, respectively.
Group V–VII HFHC plus AO1, HFHC plus AO2, and HFHC plus AO3; all MetS rats induced by HFHC in these groups were orally given the functional ingredient (AO) at the doses of 1, 10, and 100 mg/kg BW, respectively.

MetS rats in groups II–group VII were induced by a 12-week HFHC feeding. The rats that showed the characteristics of MetS were selected for further study. All assigned substances were orally administered via a gastric gavage needle once daily at period of 14 days. Then rats were assessed for anxiety, depression, and spatial and non-spatial memory, together with open field tests, after the single administration and at 7 and 14 days of treatment. To determine the possible underlying mechanisms, the assessment of oxidative stress status including brain levels of malondialdehyde and the activities of superoxide dismutase (SOD), catalase (CAT), and glutathione peroxidase (GSH-Px), together with the indirect assessments of GABA, monoamine, and acetylcholine transmitters in cerebral cortex and hippocampus were performed via the measurements of GABA-transaminase, monoamine oxidase (MAO), and acetylcholinesterase activities.

In the second section, we also explored the anxiolytic, hypnotic, and memory-enhancing effects of each component of the functional ingredient to identify the role of each ingredient in the novel functional ingredient.

### 2.4. Behavioral Assessments

#### 2.4.1. Anxiety-Like Behavior

Anxiety-like behaviors were assessed via an elevated plus maze (EPM) test. This apparatus consisted of two open arms (30 × 5 × 0.25 cm) and two closed arms (30 × 5 × 15 cm) emanating from a common central platform (5 × cm). Two pairs of identical arms were opposite to each other. This apparatus was elevated 40 cm above floor. Each animal was assessed by the number of an opened arm entry, and the time spent in an opened arm during a 5-min exploration time [47].

#### 2.4.2. Spatial Memory Evaluation

In this experiment, spatial memory was evaluated via associative learning regarding the association between the animal’s location and the platform’s location by using the external cues as guidelines for navigation to the hidden platform. A circular water bathtub (170 cm in diameter and 40 cm in depth) comprising 4 quadrants (N, S, E, and W) was filled with water at a temperature of 25.0 ± 0.5 °C, and the water surface was covered with non-toxic powder. The removable platform was submerged under water in the center of one quadrant, and the location of the platform was invisible. Each animal was trained to find the location of the platform at the same period of the day for 4 consecutive days. If it failed to locate the platform within 60 s, it was gently placed on the platform by the researcher and allowed to stay there for 30 s. The time spent navigating to the platform location was regarded as escape latency. Then, an animal was re-exposed to the same condition except that the immersed platform was removed. The time that each rat spent swimming in the quadrant previously containing the immersed platform was recorded as retention time. The test was performed by a trained researcher with a double-blind manner [48].

#### 2.4.3. Antidepressant Effect Evaluation

The antidepressant activity of the tested substances was evaluated by using forced swimming test (FST), a validated method for assessing antidepressant activity of a tested substance in an animal model. A glass cylindrical tank (30 × 20 × 15 cm^3^) was filled with tap water at height of 24 cm. Water temperature was maintained at 25 ± 3 °C on the tested day, the determination of antidepressant activity was determined by measuring immobilization time, swimming time, and climbing time within a 5-min test by a trained researcher who was blinded to the treatment [49].

#### 2.4.4. Sleep Assessment

Sleep assessment was performed by injecting pentobarbital at a dose of 50 mg/kg BW via intraperitoneal route 1 h after the administration of the tested substance. Then, the sleep onset with a criterion of a loss of righting reflex over 5 min when an animal was placed on its back was observed. The sleep latency or the duration time from the pentobarbital injection to the loss of righting reflex, and the sleeping time or the time from the loss of righting reflex to recovery, were recorded [50].

#### 2.4.5. The Open Field Test

Locomotor and exploratory activity of animals was assessed by using an open field test. A box (72 × 72 × 36 cm) with 16 squares (18 × 18 cm) on the floor was used as a tool for an assessment. A central square was drawn in the middle of the open field [51]. The animals were placed individually in the box. The number of rearing, grooming, line crossing, and center square entries were counted for 5 min. Diazepam was used as standard drug.

### 2.5. Biochemical Assays

#### 2.5.1. GABA-Transaminase Activity Assessment

Brain was isolated and homogenized with buffer (Triton X-100, 0.5%; 5 mM dithiothreitol; 1 mM pyridoxal phosphate, and 10 mM sodium phosphate buffer, pH 7.0). Then, the homogenate was centrifuged at 2000× *g* for 20 min at 0 °C. The supernatant was harvested and used for GABA-transaminase determination. In brief, 200 µL of brain tissue was mixed with 800 µL of an assay buffer consisting of GABA (20 mM), α-ketoglutarate (10 mM), and NAD (0.5 mM) in sodium phosphate buffer (0.05 M, pH 8.0), and incubated for 30 min at 21 °C. The formation of NADH was measured at 310 nm within 90 min [52]. The data were expressed as units/mg protein.

#### 2.5.2. Determination of Monoamine Oxidase (MAO) Activity

MAO activity was measured according to the protocol described previously [53]. An aliquot of brain homogenate at a volume of 50 μL was incubated with a mixture of 50 μL of chromogenic solution, and 200 μL of 500 μM of tyramine for 30 min at room temperature. Then, an absorbance was recorded at 490 nm. The activity of MAO was expressed as U/mg protein.

#### 2.5.3. Acetylcholinesterase (AChE) Activity Assessment

AChE activity assessment in brain homogenate was carried out as described [54]. A reaction mixture containing 200 μL of 0.1 mM sodium phosphate buffer (pH 8.0) and 10 μL of 0.2 M DTNB (5,5′-dithiobis(2-nitrobenzoic acid) was mixed with 20 μL of issue homogenate, and subjected to a 25 °C incubation for 5 min. At the end of incubation period, 10 μL of 15 mM acetylcholine thiochloride (ACTI) was added and incubated for 3 min. An absorbance at 412 nm was measured by using a microplate reader, and the activity of AChE was expressed as nmol/min·mg.

#### 2.5.4. Assessment of Oxidative Stress Parameters

Parameters indicating oxidative stress status including the level of malondialdehyde (MDA), and the activities of the main scavenger enzymes such as superoxide dismutase (SOD), catalase (CAT), and glutathione peroxidase (GSH-Px), were also determined. MDA level and the activities of SOD, CAT, and GSH-Px in the brain tissue were assessed according to the methods previously described by Wattanathorn et al. [54].

### 2.6. Statistical Analysis

All data are expressed as mean ± standard error of mean (SEM). Statistical significance was evaluated by using one-way analysis of variance (ANOVA), followed by the post hoc (Tukey) test by using SPSS version 21.0 (IBM Corp. Released 2012. IBM SPSS Statistics for Windows). Statistical significance was regarded at *p*-values < 0.05.

## 3. Results

### 3.1. Anxiolytic Effect

**Figure 1:**
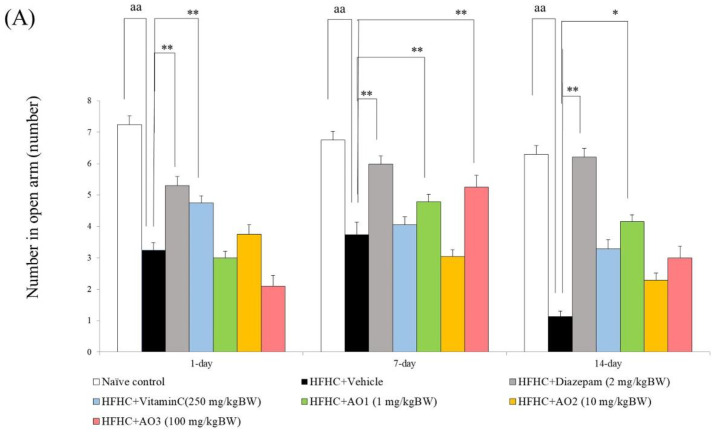

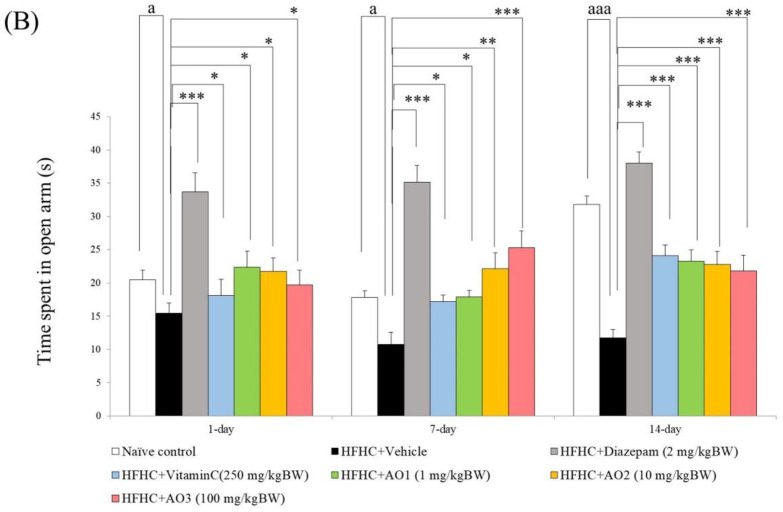
Anxiolytic effect of the functional ingredient containing cashew leaf extract and cashew apple pomace-derived dietary fiber (AO) assessed by using elevated plus maze test. (**A**) Number of open arm entries. (**B**) Time spent in an opened arm. Data are presented as mean ± SEM (*n* = 6/group). ^a^, ^aa^, ^aaa^ *p*-value < 0.05, 0.01, and 0.001, respectively; compared to naïve control that received ND and vehicle and *, **, *** *p*-value < 0.05, 0.01, and 0.001, respectively, compared to metabolic syndrome rats induced by HFHC and received vehicle.

An anxiolytic effect of AO was measured by using an elevated plus maze, a validated and widely-used tool for anxiolytic assessment of pharmacological agents [34]. Data obtained from ANOVA reveal the significant differences in the number of open arm entries among the groups on day 1, day 7, and day 14 (F(7,35) = 52.23, *p*-value < 0.001, F(7,35) = 56.47, *p*-value < 0.001, and F(7,35) = 81.89 and *p*-value < 0.001, respectively). When compared to the naïve control, HFHC-treated rats significantly decreased the number of open arm entries on day 1, day 7, and day 14 (*p*-value < 0.01 all) as shown in Figure 1A. These changes were mitigated by diazepam, a standard antianxiety drug, which served as a positive control in this part (*p*-value < 0.01 all; compared to the HFHC + vehicle group). Vitamin C also mitigated the number of opened arm entries on day 1 of treatment (*p*-value < 0.01; compared to the HFHC + vehicle group). It was found that at 7 days of treatment, AO at a dose of 100 mg/kg BW significantly increased the number of open arm entries (*p*-value < 0.05; compared to the HFHC + vehicle group). When the treatment was prolonged to 14 days, a significant increase in this parameter was observed only in the HFHC-treated rats that received AO at a dose of 1 mg/kg BW (*p*-value < 0.05; compared to the HFHC + vehicle group). The effect of AO on the time spent in an open arm is shown in Figure 1B. It was found that the current data also demonstrated a significant difference in this parameter among the groups on day 1, day 7, and day 14 (F(7,35) = 102.81, *p*-value < 0.001, F(7,35) = 125.21, *p*-value < 0.001 and F(7,35) = 104.95, *p*-value < 0.001, respectively). When compared to the naïve control, HFHC-treated rats that received the vehicle showed a reduction in the time spent in an open arm on day 1, day 7, and day 14 of the vehicle treatment (*p*-value < 0.05, 0.05, and 0.001, respectively). Diazepam, and vitamin C mitigated the change just mentioned throughout the study period (*p*-value < 0.001 all; compared to the HFHC + vehicle group). The mitigation effect of vitamin C was also observed on day 1, day 7, and day 14 of treatment (*p*-value < 0.05, 0.05, and 0.001, respectively; compared to the HFHC + vehicle group). Interestingly, AO at the doses of 1, 10 and 100 mg/kg BW significantly attenuated the reduction in time spent in an open arm induced by the HFHC diet after a single dose of administration, and at 7 and 14 days after AO administration (*p*-value < 0.05 all; *p*-value < 0.05, 0.01, and 0.001, respectively; *p*-value < 0.001 all; compared to the HFHC + vehicle group).

### 3.2. Sedative and Hypnotic Effect

**Figure 2:**
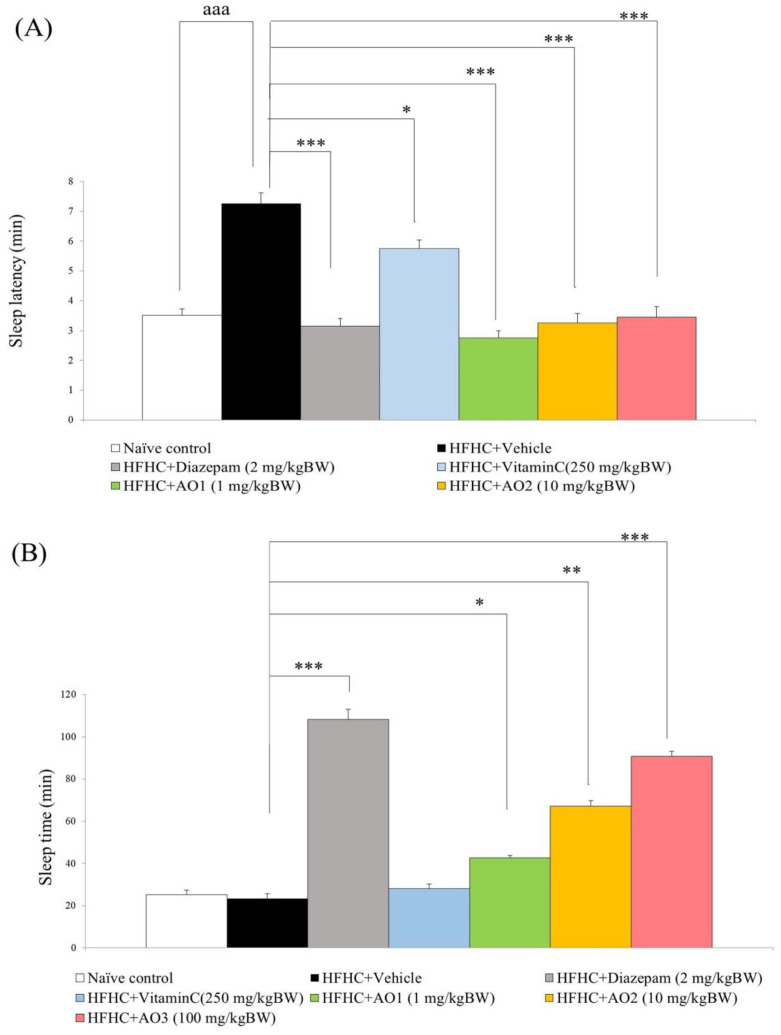
Effect of the functional ingredient containing cashew leaf extract and cashew apple pomace-derived dietary fiber (AO) on sleep assessed by using pentobarbital potentiation test. (**A**) Sleep latency. (**B**) Sleep time. Data are presented as mean ± SEM (*n* = 6/group). ^aaa^ *p*-value < 0.001, compared to naïve control that received ND and vehicle. *, **, *** *p*-value < 0.05, 0.01, and 0.001, respectively; compared to metabolic syndrome rats induced by HFHC and received vehicle.

The sedative and hypnotic effect of AO was assessed by determining the potentiation effect of AO on pentobarbital-induced sleeping behaviors. Our data showed that significant differences in sleep latency and sleep time among groups were observed (F(7,35) = 152.04), *p*-value < 0.00 and F(7,35) = 186.24, *p*-value < 0.001, respectively). HFHC-treated rats that received the vehicle had increased sleep latency (*p*-value < 0.001; compared to naïve control) but failed to produce a significant change in their sleep time as shown in Figure 2A,B. Diazepam and vitamin C significantly decreased sleep latency (*p*-value < 0.001, 0.05; compared to HFHC-treated rats + vehicle), but a significant increase in sleep time was observed only in HFHC-treated rats that received diazepam (*p*-value < 0.001; compared to HFHC-treated rats + vehicle). AO at the doses of 1, 10, and 100 mg/kg BW also produced a significant reduction in sleep latency (*p*-value < 0.001 all; compared to HFHC-treated rats + vehicle). In addition, they also increased sleep time (*p*-value < 0.05, 0.01, and 0.001, respectively; compared to HFHC-treated rats + vehicle).

### 3.3. Antidepression Effect

**Figure 3:**
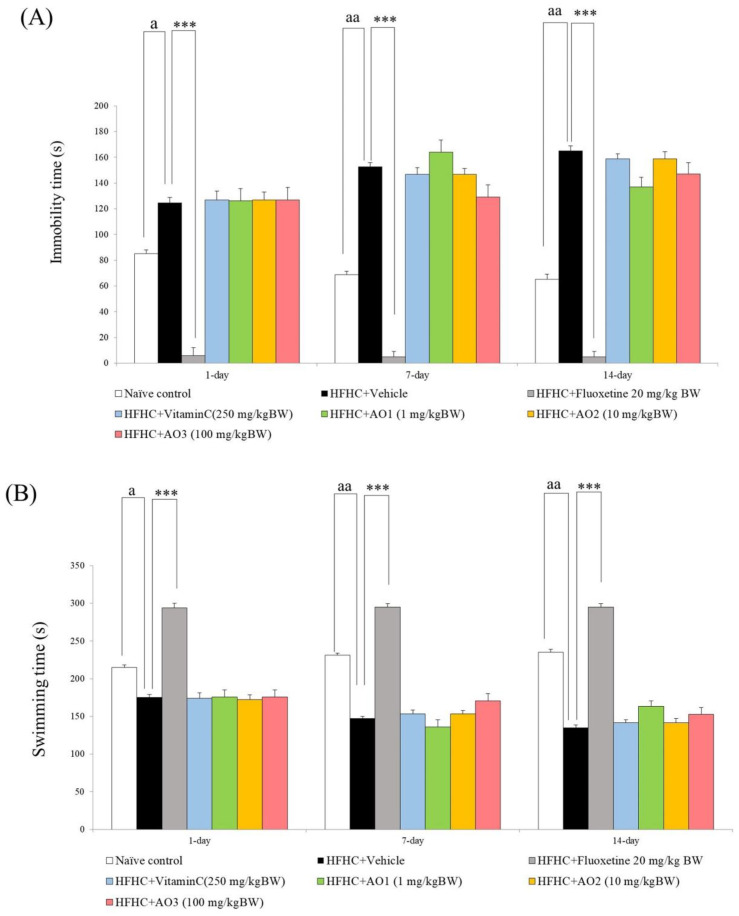

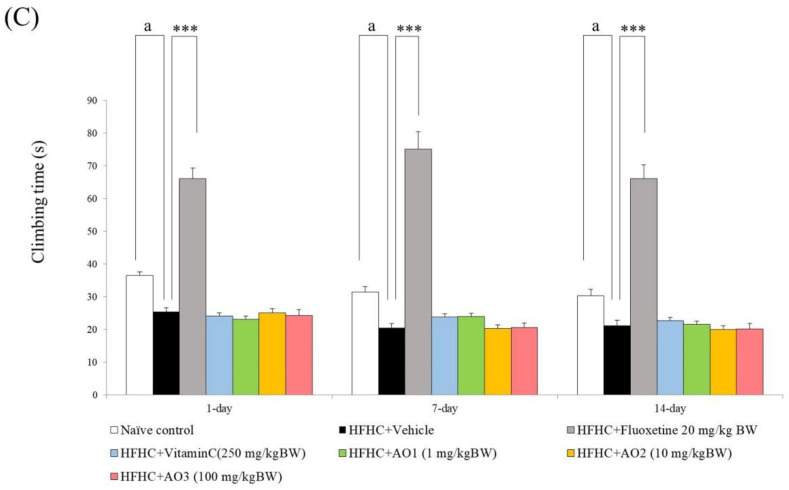
Antidepression-like activity of the functional ingredient containing cashew leaf extract and cashew pomace-derived dietary fiber (AO) evaluated by using forced swimming test. (**A**) Immobility time. (**B**) Swimming time. (**C**) Climbing time. Data are presented as mean ± SEM (*n* = 6/group). ^a^, ^aa^ *p*-value < 0.05 and 0.01 respectively, compared to naïve control that received ND and vehicle. *** *p*-value < 0.001 all; compared to metabolic syndrome rats induced by HFHC and received vehicle.

The antidepression effect of AO was also evaluated, and results are shown in Figure 3A,C. Data obtained from ANOVA analysis revealed that on day 1, day 7, and day 14, significant differences were observed among the groups in swimming (F(7,35) = 78.02, *p*-value < 0.001, F(7,35) = 98.24, *p*-value < 0.001, and F(7,35) = 81.42, *p*-value < 0.001, respectively), immobility ((F(7,35) = 72.12, *p*-value < 0.001, F(7,35) = 65.23, *p*-value < 0.001, and F(7,35) = 55.07, *p*-value < 0.001, respectively), and climbing (F(7,35) = 56.35, *p*-value < 0.001, F(7,35) = 59.74, *p*-value < 0.001, and F(7,35) = 71.09, *p*-value < 0.001, respectively). Our data revealed that when compared to the naïve control, HFHC-treated rats had increased immobility time on day 1, day 7, and day 14 (Figure 3A; *p*-value < 0.05, 0.01, and 0.01), but the swimming and climbing times decreased throughout the study period (Figure 3B; *p*-value < 0.05, 0.01, and 0.01, compared to naïve control; and Figure 3C; *p*-value < 0.05 all, compared to naïve control). Fluoxetine significantly mitigated all changes in the parameters just mentioned (*p*-value < 0.001 all; compared to HFHC + vehicle). There were no significant changes in the aforementioned parameters in HFHC-treated rats that received vitamin C, and all doses of AO.

### 3.4. Memory Enhancing Effect

**Figure 4:**
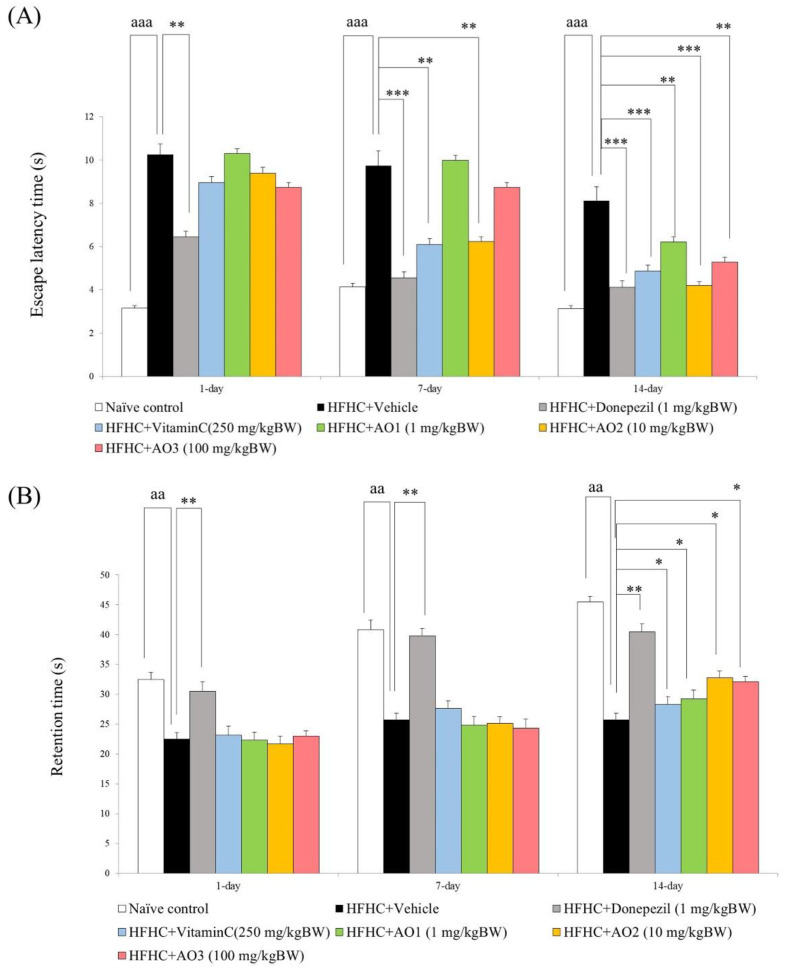
Memory-enhancing effect of the functional ingredient containing cashew leaf extract, and cashew pomace-derived dietary fiber (AO) evaluated by using Morris water maze test. (**A**) Escape latency. (**B**) Retention time. Data are presented as mean ± SEM (*n* = 6/group). ^aa^, ^aaa^ *p*-value < 0.01, and 0.001, respectively, compared to naïve control that received ND and vehicle. *, **, *** *p*-value < 0.05, 0.01, and 0.001, respectively; compared to metabolic syndrome rats induced by HFHC and received vehicle.

The present results showed that on day 1, day 7, and day 14, significant differences were observed among the groups in escape latency (F(7,35) = 132.03, *p*-value < 0.001, F(7,35) = 146.75, *p*-value < 0.001, and F(7,35) = 131.08, *p*-value < 0.001, respectively) and retention time (F(7,35) = 122.03, *p*-value < 0.001, F(7,35) = 154.74, *p*-value < 0.001, and F(7,35) = 214.24, *p*-value < 0.001, respectively). Figure 4A,B demonstrate that HFHC-treated rats revealed a significant increase in escape latency on day 1, day 7, and day 14 (*p*-value < 0.001 all; compared to naïve control) together with a decrease in the retention time (*p*-value < 0.01 all; compared to naïve control). Donepezil significantly decreased escape latency in HFHC-treated rats on day 1, day 7, and day 14 of treatment (*p*-value < 0.01, 0.001, and 0.001, respectively; compared to HFHC + vehicle). A significant reduction in the escape latency of HFHC-treated rats that received vitamin C was observed on day 7 and day 14 of treatment (*p*-value < 0.01 and 0.001, respectively; compared to HFHC + vehicle). On day 7 of treatment, a significant reduction in escape latency was observed in HFHC-treated rats that received AO at a dose of 10 mg/kg BW (*p*-value < 0.01; compared to HFHC + vehicle). However, when the treatment was extended to 14 days of treatment, AO at doses of 1, 10, and 100 mg/kg BW produced a significant reduction in the escape latency of the HFHC-treated rats (*p*-value < 0.01, 0.001, and 0.01; compared to HFHC + vehicle). Figure 4A,B show that donepezil significantly enhanced the retention time of HFHC-treated rats throughout the experimental period (*p*-value < 0.01 all; compared to HFHC + vehicle), whereas vitamin C produced a significant reduction in this parameter only on day 14 of treatment (*p*-value < 0.05; compared to HFHC + vehicle). The significant elevation in retention time was also observed in HFHC-treated rats that received AO at all doses used in this study on day 14 of treatment (*p*-value < 0.05 all; compared to HFHC + vehicle).

### 3.5. Locomotor Motor Activity

To prevent confounding errors from false positives of the behavioral tests that involved motor activity, the effects of AO on motor activity were also monitored, and the results are shown in Table 1, Table 2, Table 3 and Table 4. It was found that there were no significant changes in the number of line crossings (crossing the square boundaries with both forepaws), the number of central square area entries, the amount of rearing, and the amount of grooming in any groups when compared to the naïve intact control.

### 3.6. Neurotransmitter Changes

**Figure 5:**
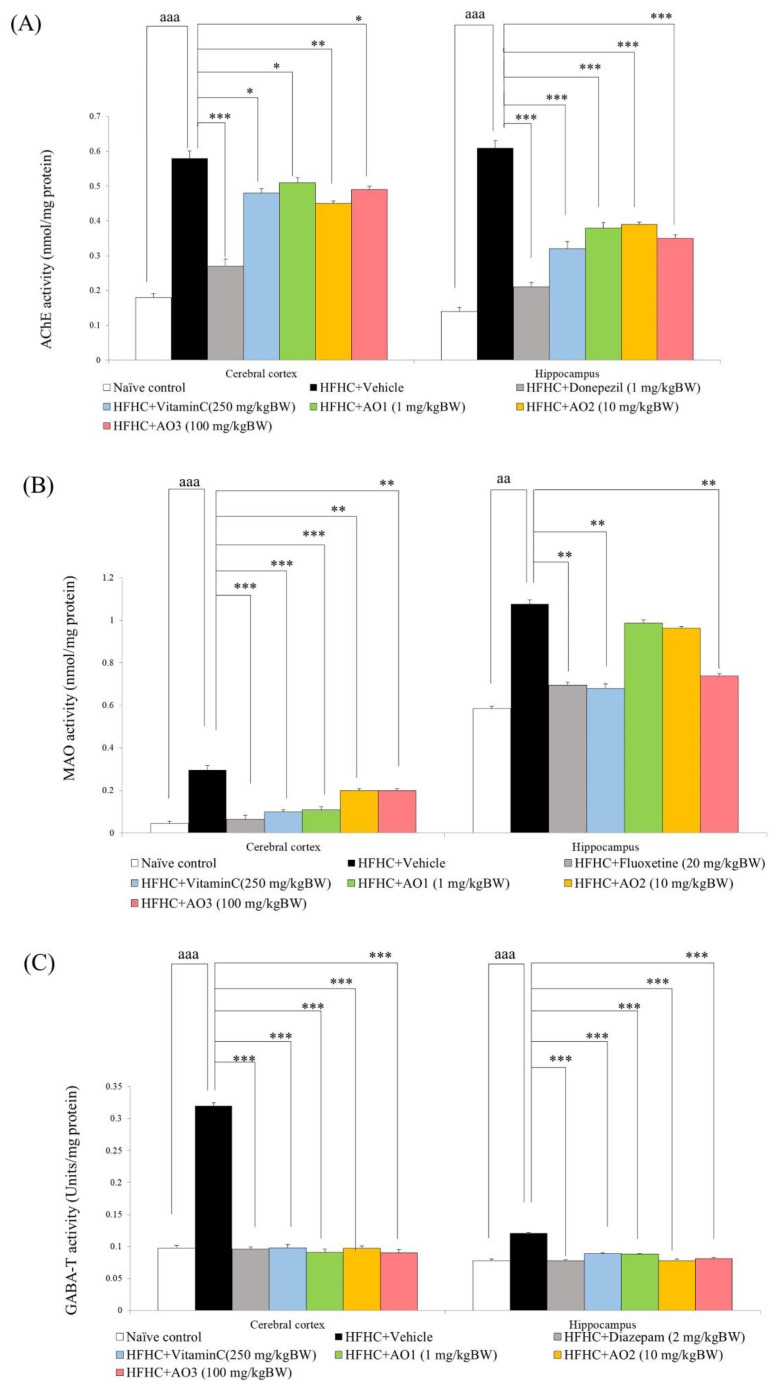
Neurotransmitter changes in cerebral cortex and hippocampus of various treatment groups assessed indirectly via the suppression of inactivation enzymes. (**A**) AChE suppression effect. (**B**) MAO suppression effect. (**C**) GABA-T suppression effect. Data are presented as mean ± SEM (*n* = 6/group). ^aa, aaa^ *p*-value < 0.01 and 0.001, respectively; compared to naïve control that received ND and vehicle. *, **, *** *p*-value < 0.05, 0.01, and 0.001, respectively; compared to metabolic syndrome rats that received HFHC and vehicle.

Figure 5A–C demonstrate the changes in acetylcholine, monoamine, and GABA in the cerebral cortex and hippocampus indirectly via the suppression of inactivation enzymes such as AChE, MAO, and GABA-T. It was demonstrated that significant differences in AChE in the cerebral cortex and hippocampus were observed (F(7,35) = 69.24, *p*-value < 0.001, F(7,35) = 84.32 and *p*-value < 0.001, respectively). There was a significant difference in MAO in both the cerebral cortex and hippocampus (F(7,35) = 86.01, *p*-value < 0.001 and F(7,35) = 95.14 and *p*-value < 0.001, respectively). The difference in GABA-T activity among groups in both areas mentioned earlier was also presented (F(7,35) = 186.35, *p*-value < 0.001 and F(7,35) = 161.55 *p*-value < 0.001, respectively). When compared to the naïve control, metabolic syndrome rats showed significant elevations in AChE, MAO, and GABA-T in the cerebral cortex and hippocampus (*p*-value < 0.001 all; compared to naïve control). The elevations in AChE in the cerebral cortex and hippocampus of metabolic syndrome rats were mitigated by donepezil and vitamin C (*p*-value < 0.001 all; *p*-value < 0.05 and 0.001; compared to the HFHC + vehicle group). AO at doses of 1, 10, and 100 mg/kg BW also significantly decreased AChE in the cerebral cortex and hippocampus (*p*-value < 0.05, 0.01, and 0.05; *p*-value < 0.001 all; compared to the HFHC + vehicle group) as shown in Figure 5A.

An increase in MAO activity in metabolic syndrome rats was also attenuated by fluoxetine and vitamin C (*p*-value < 0.001 all; *p*-value < 0.01 all; compared to the HFHC + vehicle group). AO at doses of 1 and 10 mg/kg BW produced a significant reduction in MAO only in the cerebral cortex of metabolic syndrome rats (*p*-value < 0.001 and 0.01; compared to the HFHC + vehicle group), whereas AO at a dose of 100 mg/kg BW produced a significant reduction in this parameter both in the cerebral cortex and in the hippocampus (*p*-value < 0.01 and 0.05; compared to the HFHC + vehicle group) as shown in Figure 5B.

Figure 5C reveals that the elevation in GABA-T in the cerebral cortex and hippocampus was attenuated by diazepam, vitamin C, and all doses of AO used in this study (*p*-value < 0.001 all; compared to the HFHC + vehicle group).

### 3.7. Oxidative Stress Changes

Table 5 and Table 6 reveal that metabolic syndrome rats induced by HFHC had significantly decreased SOD, CAT, and GSH-Px but increased MDA in the cerebral cortex and hippocampus (*p*-value < 0.001 all; compared to naïve control). Vitamin C, a well-known antioxidant, significantly increased SOD and GSH-Px, (*p*-value < 0.001, 0.05; *p*-value < 0.01, 0.05; compared to HFHC + vehicle) but decreased the MDA level in both the cerebral cortex and hippocampus (*p*-value < 0.001, 0.05; compared to HFHC + vehicle). No significant change in CAT activity was observed. When compared to the metabolic syndrome rats that received the vehicle, AO at doses of 1, 10, and 100 mg/kg BW increased SOD, CAT, and GSH-Px in the cerebral cortex (*p*-value < 0.001 all; *p*-value < 0.01 all; *p*-value < 0.01, 0.001, and 0.001). However, only the medium and high doses of AO significantly increased SOD, CAT, and GSH-Px activities in the hippocampus (*p*-value < 0.05, 0.001; *p*-value < 0.01 all; *p*-value < 0.05, 0.001). Furthermore, all doses of AO decreased the MDA level in the cerebral cortex (*p*-value < 0.001 all; compared to HFHC + vehicle). However, a significant reduction in MDA in the hippocampus was observed only in the metabolic syndrome rats that received either a medium or high dose of AO (*p*-value < 0.05 all; compared to HFHC + vehicle).

### 3.8. Behavioral Effects of the Leaf Extract, Fruit Pomace-Derived Dietary Fiber from A. occidentale, and the Functional Ingredient Containing the Leaf Extract and Dietary Fiber from Fruit Pomace from A. occidentale

We also determined the effect of the leaf extract, fruit pomace-derived dietary fiber from *A. occidentale*, and the functional ingredient containing the leaf extract and dietary fiber from the fruit pomace from *A. occidentale* to explore whether the observed effects can be attributed to the synergistic effect of the ingredients according to our hypothesis. Table 7 shows that the leaf extract or fruit pomace-derived dietary fiber from *A. occidentale* only improved anxiety-like behavior. However, this effect was enhanced with the functional ingredient that contained both the leaf extract and the fruit pomace from *A. occidentale* or AO. AO also exhibited memory-enhancing effect as shown in Table 7. Furthermore, AO exhibited sedative and hypnotic effects by enhancing sleep duration and decreasing sleep latency as shown in Table 8.

### 3.9. Changes in Biochemical Parameters

The effects of the leaf extract, fruit pomace-derived dietary fiber from *A. occidentale*, and the functional ingredient containing the leaf extract and dietary fiber from the fruit pomace from *A. occidentale* on biochemical parameters, including oxidative stress markers, and the suppression activities of AChE, MAO, and GABA-T were also investigated, and the results are shown in Table 9. It was clearly shown that the leaf extract and the fruit pomace-derived dietary fiber alone could produce a significant increase in SOD and a reduction in MDA in the cerebral cortex (*p*-value < 0.05 all; compared to HFHC + vehicle). However, they failed to show the positive modulation effect on other parameters investigated in this study, while AO significantly increased SOD, CAT, and GSH-Px but decreased MDA, AChE, MAO, and GABA-T in the cerebral cortex (*p*-value < 0.001, 0.001, 0.001, 0.001, 0.01, 0.01, and 0.01, respectively; compared to HFHC + vehicle). All of the aforementioned parameters, except the suppression of MAO, also showed a significant change in the hippocampus of metabolic syndrome rats that received AO at a dose of 10 mg/kg BW (*p*-value < 0.05, 0.01, 0.05, 0.05, 0.001, and 0.001, respectively; compared to HFHC + vehicle). Therefore, these data suggest that the changes in the biochemical parameters observed in this study are most likely to occur as the result of the synergistic effect of the leaf extract and the fruit pomace-derived dietary fiber from *A. occidentale.*

## 4. Discussion

Currently, anxiety, insomnia, depression, and memory impairment are the most commonly found psychiatric disorders. Despite their increasing prevalence, and the great impact of this on the socio-economic burden, treatment efficacy is still limited, and focus has been on the prevention strategy. The current study clearly demonstrates that AO exhibits anxiolytic, sleep induction, and memory-enhancing effects in metabolic syndrome rats. It also improves cholinergic, monoaminergic, and GABAergic functions together with the reduction in oxidative stress status in a rat model of metabolic syndrome induced by an HFHC diet.

Since the behavioral changes in the animals monitored according to the commonly-used neuropharmacological tests used in this study were under the influences of the effects of both motor functions and psychological functions, it is necessary to clearly demonstrate that the observed effects were not false positives due to the positive modulation effects of the tested substance on the motor function [55,56,57]. Therefore, the locomotor function was also assessed to confirm the observed changes occurred as a result of the positive modulation effect on the psychological functions of the brain. According to our results, no positive modulation effect on motor function was observed. These results suggest that the specific effect of the tested substance was on the brain areas playing important roles in psychological functions, particularly the higher brain functions such as mood regulation, learning and memory, and sleep. The data indicate the strength and validity of the study in that there was no confounding error from the modification effect on the motor system, which in turn induces false positive results.

Accumulative evidence has demonstrated that oxidative stress damage plays a crucial role on the onset and progression of psychiatric disorders such as anxiety [58] and memory [59]. In addition, excess oxidative stress induced by mitochondrial dysfunction is reported to be associated with sleep disturbances such as fatal familial insomnia [60] and sleep deprivation [61,62]. These symptoms can be improved by antioxidants. It has been revealed that an antioxidant exhibits anxiolytic action but fails to suppress depression [63]. In addition, it improves memory and oxidative-inflammatory damage in the rat hippocampus with metabolic syndrome [64]. These findings correspond with our data, which clearly demonstrate that metabolic syndrome rats that received AO, which suppresses oxidative stress products such as MDA, also had improved levels of anxiety, memory, and oxidative stress in the hippocampus, and cerebral cortex. Therefore, the anxiolytic effect and memory-enhancing effect observed in this study may occur partly via the suppression effect of AO on oxidative stress. Furthermore, it has been demonstrated that after a single administration, Vitamin C shows a significant increase in open arm entry in an elevated plus maze test, whereas AO fails to show the significant change in this parameter at this time point of assessment. These observed changes do not correspond with the improvement in oxidative stress status because the assessment of the oxidative stress parameters was performed at the end of the study, while the significant changes observed in anxiety-like behavior was assessed after the single administration. However, the data at the final assessment of anxiety-like behavior still show the corresponding changes with all oxidative stress parameters, especially in the cerebral cortex, an area that plays a pivotal role in emotion regulation [65].

Several investigations have revealed that mental disorders are associated with the disturbances of neurotransmitters, the endogenous chemical messengers that carry and amplify brain signals [66,67]. The disturbance of monoamine transmitters plays an important role in mood disorders, and the suppression of monoamine oxidase (MAO) can improve mood disorders such as anxiety [66,67,68]. A recent finding demonstrated that monoamine oxidase inhibitor (MAOI) also exhibits a neuroprotective effect [69] and improves memory [70,71]. Obesity, one of the components of metabolic syndrome, increases MAO [72,73,74]. These data correspond with our data, which reveal that metabolic syndrome rats increase anxiety-like behavior but decrease memory performance together with the elevation in monoamine oxidase (MAO) in the cerebral cortex and hippocampus. However, these changes can be counteracted by AO, the novel functional ingredient from *A. occidentale*. Therefore, the suppression of MAO may be partly responsible for the anxiolytic and memory-enhancing effects observed in this study.

Currently, cholinergic deficiency is the most well-established neurochemical change related to memory impairment. The suppression of acetylcholinesterase (AChE), which in turn increases cholinergic function, can enhance memory [75,76]. In addition, the suppression of AChE increases sleep [77,78]. Owing to the role of the cholinergic function mentioned earlier, we also explored the alteration in AChE in both the cerebral cortex and the hippocampus. It was shown that metabolic syndrome rats increased levels of AChE in the cerebral cortex and hippocampus. However, AO mitigated these changes. Thus, the memory-enhancing effect and hypnotic effect of AO observed in this study may also occur partly via an improvement in cholinergic function in both areas just mentioned.

Due to the critical role of GABA on the central nervous system, and on the numerous health benefits including stress regulation, circadian rhythm and sleep regulation, reduction in anxiety, and memory enhancement [79], the alterations in the GABAergic function have been explored. Data obtained from the previous study demonstrated that a low level of GABA or an impaired GABA function is associated with anxiety disorder [80], sleep disorders such as insomnia [81], and impairments of spatial and working memory [82]. These findings correspond with our data, which clearly demonstrate that an elevation of GABA-T, an inactivation enzyme of GABA, which indirectly decreases the GABA level and function in the cerebral cortex and hippocampus of metabolic syndrome rats, increases anxiety like-behavior and sleep latency but decreases spatial memory performance. The suppression of GABA-T, which indirectly enhances GABAergic function in both areas mentioned earlier, in metabolic syndrome rats that received AO at the dosage range used in this study improved anxiety-like behavior, memory, and both sleep latency and duration. These data suggest that the improvement in GABAergic function via the suppression of GABA-T also partly contributes a pivotal role in the anxiolytic, hypnotic, and memory-enhancing effect of GABA.

Since our functional ingredient consists of the leaf extract and dietary fiber from the pomace of *A. occidentale* fruit, which was developed on the basis of the synergistic effect of the ingredients, we also determined the changes in the aforementioned parameters by comparing the effects of the leaf extract and the dietary fiber derived from the fruit pomace on their own (the concentrations used were equivalent to the concentrations of each ingredient presented in the medium dose of the developed functional ingredient) with the metabolic syndrome rats induced by an HFHC diet that received the vehicle, and the metabolic syndrome rats that received AO at the medium dose, which produced the optimum benefits, in order to investigate the ingredient playing the main role in the observed benefits. Interestingly, most investigated parameters in this study show the tight association with AO. The association of the leaf extract or dietary fiber alone show a closed relationship with the antianxiety effect and the alterations in MDA and SOD in the cerebral cortex. The positive modulation effect of the leaf extract shows the same pattern of action as gallic acid. Therefore, our study clearly demonstrated that the beneficial effects of AO on the mental disorders investigated in this study are associated with the synergistic effect of the polyphenolic content such as gallic acid [63,64]. Therefore, gallic acid presenting in AO plays some role in the antianxiety and antioxidant effects. The precise underlying mechanisms of the synergistic effect of polyphenols, particularly gallic acid, which serves as the highest polyphenolic compound in the leaf extract and dietary fiber in AO, are still unknown. However, the synergistic effect can occur via the modulation of the pharmacokinetic and pharmacodynamic effects of an active substance (s). Furthermore, the synergistic effect can also modify the gut microbiota, particularly *Lactobacillus* spp. and *Bifidobacterium* spp., as shown in the Supplementary Material S2 (Tables S1 and S2), which in turn generates some beneficial metabolites that can improve the disturbance of neurotransmitters such as acetylcholine, monoamine, and GABA by suppressing their inactivation enzymes via modification of the gut–brain axis [83,84]. Moreover, this modification can improve neuroinflammation and cortisol giving rise to an alleviation of anxiety [85]. This mechanism may play a role in the difference in the magnitude of response between vitamin C and AO. However, these suggestions require further investigation.

The current data have clearly revealed that metabolic syndrome induced by an HFHC diet significantly alters the balance of neurotransmitters such as acetylcholine, monoamine, and GABA resulting in anxiety, memory deficit, and sleep disorder. Furthermore, the functions of the cholinergic, monoaminergic, and GABAergic systems can also be suppressed by obesity, a component in metabolic syndrome [72,86,87], finally resulting in the disturbance of functions that are associated with the aforementioned neurotransmitters including anxiety, memory, and sleep. In addition, an elevation in body weight is associated with psychiatric disorders [72]. Therefore, alterations in the neurotransmitter balance just mentioned may be attributed partly to an anti-obesity effect. It was demonstrated that the body weight of MetS rats treated with AO also decreased during the experiment (Table S3).

Our data failed to show a dose-dependent manner. This phenomenon could have partly occurred as the result of the lack of a linear relationship between the concentrations of AO and the observed parameters. It has been demonstrated that under an in vivo situation, most biological effects failed to show a linear relationship because each activity often involves many factors [87]. This finding corresponds with the data obtained from this study. Our data also demonstrate that AO can improve the neuropharmacological functions evaluated in this study by modulating the transmitters’ functions and improving oxidative stress status. Furthermore, AO is not a pure substance, but it consists of many ingredients. This raises the possibility of interactions among various ingredients, which in turn modify the effects of the active ingredient, resulting in the non-linear relationship between the concentration of the tested substance and the observed parameter. Therefore, no dose-dependent manner was observed.

## 5. Conclusions

This study is the first study to demonstrate that metabolic syndrome significantly disturbs oxidative stress regulation, and the regulation of neurotransmitters such as ACh, monoamines, and GABA, by increasing the enzyme activities in their inactivation process such as AChE, MAO, and GABA-T, and this can lead to anxiety, sleep disturbances such as insomnia, and memory impairment. These changes can be counteracted by the novel functional ingredient from *A. occidentale* (AO). AO may possibly improve the functions of ACh, monoamine, and GABA either by a reduction in the oxidative stress status induced by the elevation of antioxidant enzymes such as SOD, CAT, and GSH-Px resulting in an increase in brain plasticity and the improvement in transmitter functions such as ACh (monoamine and GABA in the cerebral cortex and hippocampus) or by suppressing AChE, MAO, and GABA-T, the main inactivation enzymes of ACh, monoamine, and GABA. Interestingly, the beneficial effects of the developed functional ingredient occur mainly via the synergistic effect of the leaf extract and the dietary fiber derived from the fruit pomace of *A. occidentale*. However, the precise understanding about their interaction requires further investigation. The current data point out that AO, the functional ingredient consisting of the leaf extract and the fruit pomace of *A. occidentale*, is a potential candidate to protect against the most common mental disorders that coexist with metabolic syndrome, and it is worth moving forward to explore the potential of AO in terms of the more common psychiatric symptoms without metabolic syndrome. Moreover, a clinical trial is essential to confirm the beneficial effects of AO.

## Figures and Tables

**Table 1:** Effect of the functional ingredient containing cashew leaf extract and cashew apple pomace-derived dietary fiber (AO) on number of cross-line entries. Data are presented as mean ± SEM) *n* = 6/group.

Treatment Group	Number of Cross-Line Entries		
	Day 1	Day 7	Day 14
Naïve control	18.63 ± 0.102	16.49 ± 0.114	0.885 ± 1.06
HFHC + Vehicle	20.50 ± 0.125	15.13 ± 0.124	0.852 ± 0.97
HFHC + Vitamin C (250 mg/kgBW)	19.70 ± 1.51	16.28 ± 0.135	0.979 ± 0.91
HFHC + Diazepam (2 mg/kgBW)	18.25 ± 1.22	14.25 ± 0.105	0.1055 ± 0.98
HFHC + Fluoxetine 20 mg/kg BW	18.15 ± 1.25	0.1565 ± 0.112	0.954 ± 0.101
HFHC + Donepezil (1 mg/kg BW)	17.33 ± 1.45	14.38 ± 0.103	0.1002 ± 0.102
HFHC + AO1 (1 mg/kgBW)	18.05 ± 0.114	14.29 ± 0.092	0.1045 ± 0.090
HFHC + AO2 (10 mg/kgBW)	18.16 ± 0.112	15.11 ± 0.103	0.990 ± 1.16
HFHC + AO3 (100 mg/kgBW)	20.54 ± 0.154	15.13 ± 0.96	0.954 ± 0.102
F-test, *p*-value	F(3,51) = 1.04, *p*-value = 0.191	F(3,51) = 1.56, *p*-value = 0.541	F(3,51) = 0.94, *p*-value = 0.981

**Table 2:** Effect of the functional ingredient containing cashew leaf extract and cashew apple pomace-derived dietary fiber (AO) on number of central square entries. Data are presented as mean ± SEM) *n =* 6/group.

Treatment Group	Number of Central Square Entries		
	Day 1	Day 7	Day 14
Naïve control	2.25 ± 0.42	1.85 ± 0.29	0.63 ± 0.06
HFHC + Vehicle	2.13 ± 0.25	1.75 ± 0.27	0.65 ± 0.07
HFHC + Vitamin C (250 mg/kgBW)	1.97 ± 0.51	1.61 ± 0.35	0.76 ± 0.01
HFHC + Diazepam (2 mg/kgBW)	1.95 ± 0.25	1.75 ± 0.31	0.75 ± 0.08
HFHC + Fluoxetine 20 mg/kg BW	1.87 ± 0.45	1.65 ± 0.12	0.64 ± 0.11
HFHC + Donepezil (1 mg/kg BW)	2.05 ± 0.35	1.80 ± 0.23	0.75 ± 0.02
HFHC + AO1 (1 mg/kgBW)	2.15 ± 0.74	1.63 ± 0.22	0.54 ± 0.08
HFHC + AO2 (10 mg/kgBW)	2.25 ± 0.42	1.54 ± 0.33	0.63 ± 0.05
HFHC + AO3 (100 mg/kgBW)	1.93 ± 0.54	1.74 ± 0.26	0.72 ± 0.02
F-test, *p*-value	F(3,51) = 0.84, *p*-value = 0.997	F(3,51) = 1.86, *p*-value = 0.673	F(3,51) = 1.84, *p*-value = 0.745

**Table 3:** Effect of the functional ingredient containing cashew leaf extract and cashew apple pomace-derived dietary fiber (AO) on number of rearing events. Data are presented as mean ± SEM) *n =* 6/group.

Treatment Group	Number of Rearing Events		
	Day 1	Day 7	Day 14
Naïve control	0.63 ± 0.15	0.55 ± 0.014	0.54 ± 0.016
HFHC + Vehicle	0.61 ± 0.17	0.63 ± 0.014	0.52 ± 0.17
HFHC + Vitamin C (250 mg/kgBW)	0.58 ± 0.12	0.60 ± 0.15	0.48 ± 0.14
HFHC + Diazepam (2 mg/kgBW)	0.59 ± 0.25	0.64 ± 0.11	0.055 ± 0.18
HFHC + Fluoxetine 20 mg/kg BW	0.57 ± 0.21	0.53 ± 0.012	0.054 ± 0.013
HFHC + Donepezil (1 mg/kg BW)	0.61 ± 0.18	0.57 ± 0.11	0.51 ± 0.017
HFHC + AO1 (1 mg/kgBW)	0.60 ± 0.017	0.57 ± 0.22	0.055 ± 0.018
HFHC + AO2 (10 mg/kgBW)	0.71 ± 0.22	0.62 ± 0.023	0.57 ± 0.016
HFHC + AO3 (100 mg/kgBW)	0.68 ± 0.22	0.63 ± 0.21	0.58 ± 0.014
F-test, *p*-value	F(3,51) = 1.20, *p*-value = 0.573	F(3,51) = 1.16, *p*-value = 0.475	F(3,51) = 1.75, *p*-value = 0.486

**Table 4:** Effect of the functional ingredient containing cashew leaf extract and cashew apple pomace-derived dietary fiber (AO) on number of grooming events. Data are presented as mean ± SEM) *n =* 6/group.

Treatment Group	Number of Grooming Events		
	1-Day	7-Day	14-Day
Naïve control	2.38 ± 0.34	2.65 ± 0.31	2.50 ± 0.62
HFHC + Vehicle	2.34 ± 0.25	2.25 ± 0.32	2.25 ± 0.66
HFHC + Vitamin C (250 mg/kgBW)	2.58 ± 0.31	2.60 ± 0.48	2.51 ± 0.53
HFHC + Diazepam (2 mg/kgBW)	2.28 ± 0.27	2.25 ± 0.54	2.75 ± 0.53
HFHC + Fluoxetine 20 mg/kg BW	2.25 ± 0.027	2.45 ± 0.44	2.41 ± 0.51
HFHC + Donepezil (1 mg/kg BW)	2.30 ± 0.45	2.45 ± 0.46	2.46 ± 0.48
HFHC + AO1 (1 mg/kgBW)	2.13 ± 0.50	2.49 ± 0.45	2.44 ± 0.47
HFHC + AO2 (10 mg/kgBW)	2.35 ± 0.47	2.54 ± 0.50	2.48 ± 0.47
HFHC + AO3 (100 mg/kgBW)	2.25 ± 0.48	2.54 ± 0.51	2.45 ± 0.45
F-test, *p*-value	F(3,51) = 1.94, *p*-value = 0.653	F(3,51) = 0.96, *p*-value = 0.904	F(3,51) = 1.84, *p*-value = 0.32

**Table 5:** Oxidative stress markers including malondialdehyde (MDA) level, and the activities of main endogenous antioxidant enzymes such as superoxide dismutase (SOD), catalase (CAT), and glutathione peroxides (GSH-Px) in cerebral cortex.

Treatment Group	MDA Level (ng/mg.Protein)	SOD Activity (Units/mg.Protein)	CAT Activity (Units/mg.Protein)	GSH-Px Activity (Units/mg.Protein)
Naïve control	0.28 ± 0.04	7.14 ± 0.23	75.34 ± 2.51	10.14 ± 0.54
HFHC + Vehicle	1.58 ± 0.23 ^aaa^	1.25 ± 0.43 ^aaa^	23.50 ± 6.85 ^aaa^	1.48 ± 0.53 ^aaa^
HFHC + Vitamin C (250 mg/kgBW)	0.49 ± 0.02 ***	4.58 ± 0.30 ***	55.72 ± 6.74	5.03 ± 0.42 *
HFHC + AO1 (1 mg/kgBW)	0.35 ± 0.02 ***	6.26 ± 0.24 ***	62.85 ± 7.71 ***	7.79 ± 0.46 ***
HFHC + AO2 (10 mg/kgBW)	0.31 ± 0.04 ***	6.66 ± 0.74 ***	71.57 ± 8.25 ***	7.93 ± 1.14 ***
HFHC + AO3 (100 mg/kgBW)	0.31 ± 0.02 ***	6.43 ± 0.56 ***	72.73 ± 5.12 ***	7.59 ± 0.31 ***
F-test, *p*-value	F(9,27) = 204.48, *p*-value < 0.001	F(9,27) = 313.09, *p*-value < 0.001	F(9,27) = 168.22, *p*-value < 0.001	F(9,27) = 258.93, *p*-value < 0.001

Data are presented as mean ± SEM (*n* = 6/group). ^aaa^ *p*-value < 0.001 all; compared to naïve control that received ND and vehicle *, *** *p*-value < 0.05 and 0.001, respectively; compared to metabolic syndrome rats that received HFHC and vehicle.

**Table 6:** Oxidative stress markers including malondialdehyde (MDA) level, and the activities of main endogenous antioxidant enzymes such as superoxide dismutase (SOD), catalase (CAT), and glutathione peroxides (GSH-Px) in hippocampus.

Treatment Group	MDA Level (ng/mg.Protein)	SOD Activity (Units/mg.Protein)	CAT Activity (Units/mg.Protein)	GSH-Px Activity (Units/mg.Protein)
Naïve control	0.29 ± 0.02	6.49 ± 0.14	16.85 ± 1.26	7.96 ± 0.64
HFHC + Vehicle	2.33 ± 0.25 ^aaa^	1.16 ± 0.24 ^aaa^	0.52 ± 0.09 ^aaa^	0.99 ± 0.16 ^aaa^
HFHC + Vitamin C (250 mg/kgBW)	0.52 ± 0.04 *	4.38 ± 0.35 **	6.99 ± 0.61	5.79 ± 0.36 *
HFHC + AO1 (1 mg/kgBW)	0.58 ± 0.04	3.67 ± 0.43	6.25 ± 0.30	3.99 ± 0.32
HFHC + AO2 (10 mg/kgBW)	0.52 ± 0.02 *	4.09 ± 0.33 *	14.90 ± 1.36 **	5.77 ± 0.96 *
HFHC + AO3 (100 mg/kgBW)	0.51 ± 0.04 *	5.17 ± 0.86 ***	15.54 ± 3.02 **	6.54 ± 1.68 ***
F-test, *p*-value	F(9,27) = 82.03, *p*-value < 0.001	F(9,27) = 57.01, *p*-value < 0.001	F(9,27) = 80.42, *p*-value < 0.001	F(9,27) = 75.23, *p*-value < 0.001

Data are presented as mean ± SEM (*n* = 6/group). ^aaa^ *p*-value < 0.01 and 0.001, respectively; compared to naïve control that received ND and vehicle. *, **, *** *p*-value < 0.05, 0.01 and 0.001, respectively; compared to metabolic syndrome rats that received HFHC, and vehicle.

**Table 7:** Effects of the leaf extract, fruit pomace-derived dietary fiber, and the functional ingredient from *A. occidentale* on anxiolytic activity and memory-enhancing effect.

Behavior Test		Treatment Group	Day 1	Day 7	Day 14
**Elevated plus maze**	Number in open arm (number)	HFHC + Vehicle	4.25 ± 0.23	3.75 ± 0.39	1.13 ± 0.17
		HFHC + Cashew leaves	4.08 ± 0.31	2.50 ± 0.24	3.78 ± 0.25
		HFHC + Fiber	3.70 ± 0.22	2.80 ± 0.27	3.38 ± 0.27
		HFHC + AO 10 mg/kg	3.75 ± 0.30	3.05 ± 0.21	2.29 ± 0.22
	F-test, *p*-value		F(4,20) = 1.24, *p*-value = 0.087	F(4,20) = 3.11, *p*-value = 0.095	F(4,20) = 0.92, *p*-value = 0.061
	Time spent in open arm (s)	HFHC + Vehicle	15.50 ± 1.47	10.80 ± 2.02	11.80 ± 1.20
		HFHC + Cashew leaves	17.10 ± 3.21	21.16 ± 3.80 **	18.10 ± 2.57 *
		HFHC + Fiber	16.42 ± 1.37	16.75 ± 1.35 *	17.42 ± 1.97 *
		HFHC + AO 10 mg/kg	21.71 ± 2.61 *	22.15 ± 2.61 **	22.83 ± 2.58 ***
	F-test, *p*-value		F(4,20) = 14.20, *p*-value < 0.05	F(4,20) = 23.02, *p*-value < 0.01	F(4,20) = 94.16, *p*-value < 0.001
**Morris Water Maze**	Escape latency times (s)	HFHC + Vehicle	10.25 ± 0.83	9.75 ± 0.79	8.13 ± 0.47
		HFHC + Cashew leaves	8.08 ± 0.91	8.50 ± 0.84	8.78 ± 0.55
		HFHC + Fiber	9.70 ± 0.72	10.80 ± 0.87	8.34 ± 0.77
		HFHC + AO 10 mg/kg	9.45 ± 0.90	6.25 ± 0.71 **	4.21 ± 0.22 ***
	F-test, *p*-value		F(4,20) = 1.01, *p*-value = 0.105	F(4,20) = 18.36, *p*-value < 0.01	F(4,20) = 75.07, *p*-value < 0.001
	Retention times (s)	HFHC + Vehicle	22.25 ± 0.23	25.75 ± 0.39	25.83 ± 0.17
		HFHC + Cashew leaves	22.45 ± 0.31	25.50 ± 0.24	25.78 ± 0.25
		HFHC + Fiber	23.70 ± 0.42	24.80 ± 0.27	25.38 ± 0.27
		HFHC + AO 10 mg/kg	21.23 ± 0.30	25.46 ± 0.21	32.45 ± 0.22 *
	F-test, *p*-value		F(4,20) = 0.81 *p*-value = 0.905	F(4,20) = 1.16, *p*-value = 0.716	F(4,20) = 8.28, *p*-value < 0.05

Data are presented as mean ± SEM (*n* = 6/group). *, **, *** *p*-value < 0.05, 0.01, and 0.001, respectively; compared to metabolic syndrome rats that received HFHC and vehicle.

**Table 8:** Effects of the leaf extract, fruit pomace-derived dietary fiber, and the functional ingredient from *A. occidentale* on sedative and hypnotic effects.

Sedative Test	Treatment Group	Sedative Test (Min)
Sleep latency (min)	HFHC + Vehicle	7.26 ± 0.26
	HFHC + Cashew leaves	7.16 ± 0.46
	HFHC + Fiber	9.26 ± 0.26
	HFHC + AO 10 mg/kg	3.25 ± 0.27 ***
F-test, *p*-value		F(4,20) = 115.94, *p*-value < 0.001
Sleep time (min)	HFHC + Vehicle	23.33 ± 0.97
	HFHC + Cashew leaves	23.25 ± 0.12
	HFHC + Fiber	24.13 ± 0.57
	HFHC + AO 10 mg/kg	67.14 ± 0.42 ***
F-test, *p*-value		F(4,20) = 124.62, *p*-value < 0.001

Data are presented as mean ± SEM (*n* = 6/group). *** *p*-value < 0.001, compared to metabolic syndrome rats that received HFHC and vehicle.

**Table 9:** Changes in oxidative stress markers including malondialdehyde (MDA), superoxide dismutase (SOD), catalase (CAT), and glutathione peroxidase (GSH-Px), and the activities of acetylcholinesterase (AChE), monoamine oxidase (MAO), and gamma aminobutyric acid transaminase (GABA-T) in cerebral cortex and hippocampus of metabolic syndrome rats.

Area	Treatment Group	MDA Level (ng/mg.Protein)	SOD Activity (Units/mg.Protein)	CAT Activity (Units/mg.Protein)	GSH-Px Activity (Units/mg. Protein)	GABA-T Activity (Units/mg. protein)	MAO Activity (nmol/mg. Protein)	AChE Activity (nmol/mg.Protein)
Cerebral cortex	HFHC + Vehicle	1.58 ± 0.23	1.25 ± 0.43	23.50 ± 6.85	1.48 ± 0.53	0.320 ± 0.005	0.295 ± 0.005	0.580 ± 0.005
	HFHC + Cashew leaves	1.18 ± 0.12 *	3.46 ± 0.33 *	40.77 ± 3.85	3.86 ± 0.21	0.191 ± 0.005	0.291 ± 0.005	0.591 ± 0.005
	HFHC + Fiber	1.14 ± 0.12 *	3.60 ± 0.45 *	41.23 ± 3.15	4.05 ± 0.30	0.186 ± 0.004	0.287 ± 0.004	0.586 ± 0.004
	HFHC + AO 10 mg/kg	0.31 ± 0.04 ***	6.66 ± 0.74 ***	71.57 ± 8.25 ***	7.93 ± 1.14 ***	0.097 ± 0.005 **	0.199 ± 0.005 **	0.451 ± 0.005 **
F-test, *p*-value		F(4,20) = 64.74, *p*-value < 0.001	F(4,20) = 73.18, *p*-value < 0.001	F(4,20) = 94.76, *p*-value < 0.001	F(4,20) = 154.10, *p*-value < 0.001	F(4,20) = 34.54, *p*-value < 0.01	F(4,20) = 24.25, *p*-value < 0.01	F(4,20) = 14.10, *p*-value < 0.01
Hippocampus	HFHC + Vehicle	2.33 ± 0.25	1.16 ± 0.24	3.52 ± 0.09	0.99 ± 0.16	0.121 ± 0.001	1.075 ± 0.021	0.611 ± 0.001
	HFHC + Cashew leaves	2.08 ± 0.31	1.50 ± 0.24	2.78 ± 0.25	1.70 ± 0.24	0.101 ± 0.002 *	1.101 ± 0.025	0.601 ± 0.002
	HFHC + Fiber	2.70 ± 0.22	1.80 ± 0.27	2.38 ± 0.27	1.68 ± 0.21	0.117 ± 0.002	0.970 ± 0.022	0.597 ± 0.002
	HFHC + AO 10 mg/kg	0.52 ± 0.02 *	4.09 ± 0.33 *	14.90 ± 1.36 **	5.77 ± 0.96 *	0.078 ± 0.002 ***	0.968 ± 0.012	0.355 ± 0.002 ***
F-test, *p*-value		F(4,20) = 9.53, *p*-value < 0.05	F(4,20) = 10.75, *p*-value < 0.05	F(4,20) = 45.85, *p*-value < 0.01	F(4,20) = 10.84, *p*-value < 0.05	F(4,20) = 103.42, *p*-value < 0.001	F(4,20) = 1.16, *p*-value < =0.511	F(4,20) = 54.66, *p*-value < 0.001

Data are presented as mean ± SEM (*n* = 6/group). *, **, *** *p*-value < 0.05, 0.01, and 0.001, respectively; compared to metabolic syndrome rats that received HFHC and vehicle.

## Data Availability

The data presented in this study are available on request from the corresponding author. The data are not publicly available due to a trade secret and petty patent registration process.

## Supplementary Materials

The following supporting information can be downloaded at: https://www.mdpi.com/article/10.3390/antiox11112203/s1, Figure S1: The fingerprint chromatogram of AO, Table S1: The growth of *Lactobacillus* spp. Table S2: The growth of *Bifidobacterium* spp. Table S3: Body weights.
