# Supplementary material for: Anxiety, Insomnia, and Memory Impairment in Metabolic Syndrome Rats Are Alleviated by the Novel Functional Ingredients from Anacardium occidentale
Source: Antioxidants (Basel). 2022 Nov 7;11(11):2203. doi: 10.3390/antiox11112203 (PMC9686671; doi:10.3390/antiox11112203)
Supplement: Supplementary file 1 [file antioxidants-11-02203-s001.zip › antioxidants-1882559-supplementary.pdf]

antioxidants

MDPI

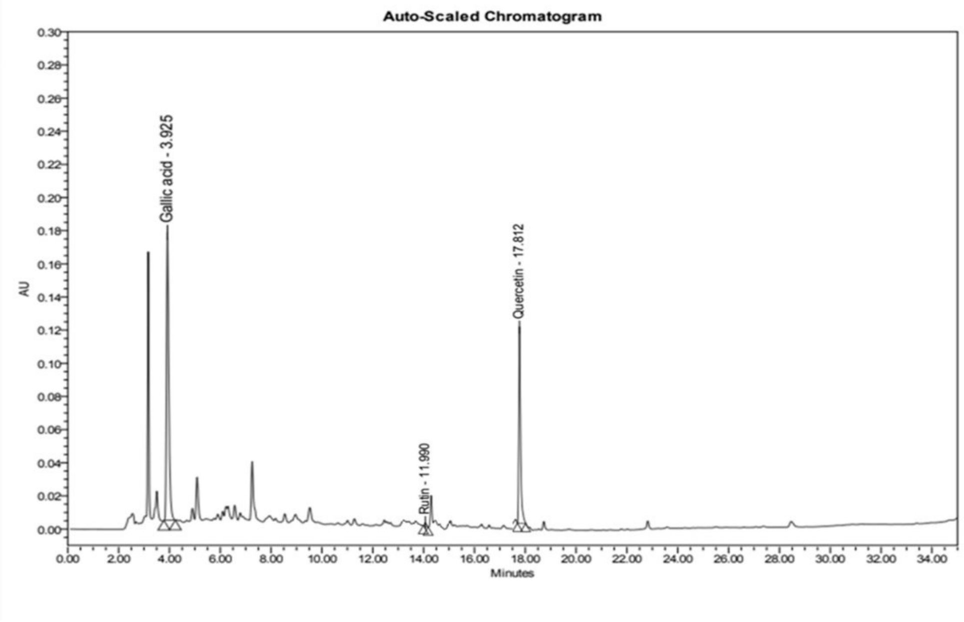

Figure S1. The fingerprint chromatogram of the cashew leaves -extract plus cashew apple pomace-derived fiber (AO). Using Purospher®STAR, C-18 encapped (5µm), LiChroCART®250-4.6 and HPLC-Cartridge, Sorbet Lot No. HX255346 (Merk, Germany) with guard column (Merk, Germany).

Table S1. Effect of the functional ingredients consisting of cashew leaf extract and a.cashew apple pomace-derived dietary fiber (AO) on the growth of *Lactobacillus spp.*

**Treatment Group**	***Lactobacillus spp*** **(%Change)**
HFHC+Vehicle	1.044±0.05
HFHC+ leaves extract	5.353±0.31
HFHC+Fiber	156.087±4.35 ***
HFHC+AO2	195.032±3.18 ***
F-test,p-value	$F(5,19) = 369.05$, p-value< 0.0001

Data are presented as mean ± SEM (n=6/group). *** p-value<0.001; compared to vehicle.

Table S2. Effect of the functional ingredients consisting of cashew leaf extract and a.cashew apple pomace-derived dietary fiber (AO) on the growth of *Bifidobacterium spp*.

**Treatment Group**	***Bifidobacterium spp.*** **(%Change)**
HFHC+Vehicle	0.411±0.02
HFHC+ leaves extract	9.594±0.27
HFHC+Fiber	38.913±1.82 ***
HFHC+AO2	52.466±1.50 ***
F-test,p-value	$F(5,19) = 253.16$, p-value< 0.001

Data are presented as mean ± SEM (n=6/group). *** p-value<0.001; compared to vehicle.

Table S3. Effect of the functional ingredient containing cashew leaves extract and cashew apple pomace-derived dietary fiber (AO) on body weights. Data are presented as mean ± SEM)n=6/group.

Treatment Group	Body Weights (g)		
	1-Day	7-Day	14-Day
Naïve control	488.25±1.23	496.75±1.12	494.25±1.23
HFHC+Vehicle	537.25±2.43 [aaa]	597.25±2.24[aaa]	598.75±2.20[aaa]
HFHC+Vitamin C (250 mg/kgBW)	537.5±2.46	525.45±2.34 ***	517.50±2.32 ***
HFHC+Diazepam (2 mg/kgBW)	536.75±2.34	606.52±2.42	599.54±3.35
HFHC+Fluoxetine 20 mg/kg BW	537.89±3.25	585.75±3.21	579.87±2.30
HFHC+Donepezil (1 mg/kg BW)	536.58±2.77	598.12±3.25	589.21±3.12
HFHC+AO1 (1mg/kgBW)	536.50±2.56	530.64±2.42 ***	544.21±3.20 ***
HFHC+AO2 (10 mg/kgBW)	536.75±2.67	542.25±2.45 ***	554.75±2.41 ***
HFHC+AO3 (100 mg/kgBW)	537.5±2.40	564.75±2.65 ***	577.25±2.34 **
F-test,p-value	F(7,47) = 2.41, p-value< 0.001	F(7,47) = 103.29, p-value< 0.001	F(7,47) = 68.43, p-value< 0.001

Data are presented as mean ± SEM (n=6/group). [aaa] p-value< 0.001, compared to naïve control which received ND and **, *** p-value< 0.01, and 0.001,.respectively; compared to metabolic syndrome rats which received HFHC and.vehicle.
